# Exploring hemodynamic measurements from the Tromsø Study for prediction of cardiovascular disease using traditional statistical models and machine learning approaches

**DOI:** 10.1038/s41598-026-57003-5

**Published:** 2026-06-13

**Authors:** Naomi Azulay, Bjørn-Jostein Singstad, Henrik Schirmer, Maja-Lisa Løchen, Roy Bjørkholt Olsen, Trond Geir Jenssen, Audun Stubhaug, Christopher Sivert Nielsen, Leiv Arne Rosseland, Christian Tronstad

**Affiliations:** 1 Department of Research and Development, Division of Emergencies and Critical Care, Oslo University Hospital, Oslo, Norway; 2 Institute of Clinical Medicine, University of Oslo, Oslo, Norway; 3 Medical Technology and E-Health, Akershus University Hospital, Lørenskog, Norway; 4 Department of Radiology, Vestfold Hospital Trust, Tønsberg, Norway; 5 Department of Cardiology, Akershus University Hospital, Lørenskog, Norway; 6 Department of Clinical Medicine, UiT The Arctic University of Norway, Tromsø, Norway; 7 Department of Cardiology, University Hospital of North Norway, UNN, Tromsø, Norway; 8 Department of Community Medicine, UiT the Arctic University of Norway, Tromsø, Norway; 9 Department of Anesthesiology and Intensive Care, Sørlandet Hospital, Arendal, Norway; 10 Department of Transplantation Medicine, Section of Nephrology, Oslo University Hospital, Rikshospitalet, Oslo, Norway; 11 Department of Pain Management and Research, Oslo University Hospital, Oslo, Norway; 12 Department of Chronic Diseases, Norwegian Institute of Public Health, Oslo, Norway; 13 Department of Clinical and Biomedical Engineering, Oslo University Hospital, Oslo, Norway

**Keywords:** Cardiology, Diseases, Health care, Medical research, Risk factors

## Abstract

In Norway, NORRISK2 is the government-recommended risk model for predicting an individual’s 10-year probability of getting cardiovascular disease (CVD). This study aims to investigate the potential for improvement of CVD prediction by using hemodynamic measurements from a non-invasive beat-to-beat blood pressure monitor, taken as part of pain sensitivity assessment with the cold-pressor test (CPT) during the Tromsø6 Study (2007–2008). Using 6694 recordings, ultra-short-term pulse rate variability (PRV) and baroreflex sensitivity (BRS) obtained during the CPT were added as additional variables into the existing NORRISK2 survival model (extended model). In addition, the time-series data was used in a machine learning (ML) model without the NORRISK2 background variables. Both models were compared to a recalibration of the original NORRISK2 model. The predictions from the recalibrated NORRISK2 model and the ML model were then combined with logistic regression. The statistical models performed similarly on the test set, with an area under the receiver operating characteristic (AUROC) of 0.8 (95% CI: 0.71–0.86), 0.79 (0.71–0.85) and 0.77 (0.69–0.84) (original, recalibrated and extended NORRISK2, respectively). The ML model using only hemodynamic measurements obtained a test set AUROC of 0.73 (0.67–0.80). Combining the NORRISK2 and ML model did not increase the AUROC. Adding ultra-short-term PRV and BRS derived from Tromsø6 did not improve the prediction of the NORRISK2 model either. Although with lower accuracy, the beat-to-beat time series of hemodynamic variables from a CPT had a significant (p < 0.01) ability to predict future CVD without any other person-specific data.

**Supplementary Information:**

The online version contains supplementary material available at https://doi.org/10.1038/s41598-026-57003-5.

## Introduction

Cardiovascular disease (CVD) is the leading cause of death globally, estimated to account for a third of all deaths in 2023^1^. While an aging and growing global population has increased the number of people affected by CVD, advancements in treatment and prevention have significantly reduced the age- and population-adjusted burden of disease^2^.

In Norway, there has been a steady decline in the number of cardiovascular deaths over the last 50 years. As a result, CVD became the second leading cause of death in 2017, when cancer deaths surpassed it^3^. Nevertheless, identifying individuals at risk and starting interventions early could further reduce CVD-related morbidity and mortality. For this reason, many countries include risk prediction in their strategy for preventing CVD. There are some well-known and much used risk calculators, like the Framingham Risk Score from the USA and SCORE2^4^ for European populations. NORRISK2^5^ is the Norwegian version, modeled on a Norwegian population in 2017. The NORRISK^6^ and NORRISK2 models were developed because both the Framingham and SCORE models overestimated the risk in the Norwegian population^6^, resulting in most men over 60 being placed in the high-risk group. NORRISK2 is a Fine-Gray survival model that predicts the 10-year risk of having a myocardial infarction (MI) or cerebral stroke based on traditional risk factors for CVD and is a well-established tool implemented by the Norwegian Directorate of Health (https://hjerterisiko.helsedirektoratet.no).

Many studies have found an association between hemodynamic variables and risk of CVD (e.g. blood pressure (BP)^7^, heart rate (HR)^8^, resting HR^9^, heart rate variability (HRV)^10^, arterial stiffness/pulse wave velocity^11,12^), but except for BP, these types of variables are not included in the established risk models mentioned above, including NORRISK2. For example, measurement of HRV is a well-known method for studying activity in the autonomic nervous system, with lower HRV indicating poor cardiac autonomic control, which may in turn contribute to an increased risk of CVD. Both HRV and baroreflex sensitivity (BRS) have been shown to be able to predict patient health outcomes such as coronary heart disease^13^, intracerebral and subarachnoid hemorrhages^14^ and acute stroke^15^.

Over the last decade, continuous HR monitoring has become more accessible, largely due to the integration of photoplethysmograpy (PPG) in wearable devices such as smartwatches. This enables not only logging of HR, but also an estimation of HRV. Due to the simplicity of the method, measurements taken with PPG have been widely studied for CVD^16^. PPG has been widely used in studies aiming to predict cardiac disease where machine learning (ML) techniques are commonly employed to develop classification and regression models. In contrast to traditional regression-based risk models, ML approaches are particularly well-suited for capturing nonlinear relationships and complex temporal dynamics in high-frequency physiological signals. Moreover, unlike conventional statistical models that rely on summary measures of physiological data, ML methods can utilize the full temporal structure of beat-to-beat hemodynamic signals. This enables the identification of subtle and dynamic patterns in cardiovascular dynamic response that may not be detectable using statistically derived variables alone. In many cases, these models have achieved classification accuracies exceeding 95% in detecting cardiac conditions^17^.

In addition to HR, smart-watch integrations of BP monitoring are emerging, for example as a miniaturized cuff-based BP wrist-watch^18^. While cuffless wearable BP measurement may not yet be ready for clinical use^19,20^, this is currently a very active research field^21^ and may provide acceptable cuffless BP measurement in smart-watches in the near future. In that case, BRS may also be estimated from these types of measurements.

This increasing availability of wearable devices capable of recording hemodynamic data provides an accessible source of information that may potentially improve CVD risk prediction. In addition to the calculated HR and BP values, other features available from the raw plethysmographic or BP waveform may be relevant in risk prediction, such as the time domain features^22^. Not only the baseline levels of these variables, but also the changes in hemodynamics due to stressors such as the cold-pressor test (CPT) may be relevant in predicting future cardiovascular events^23^.

**Fig. 1:**
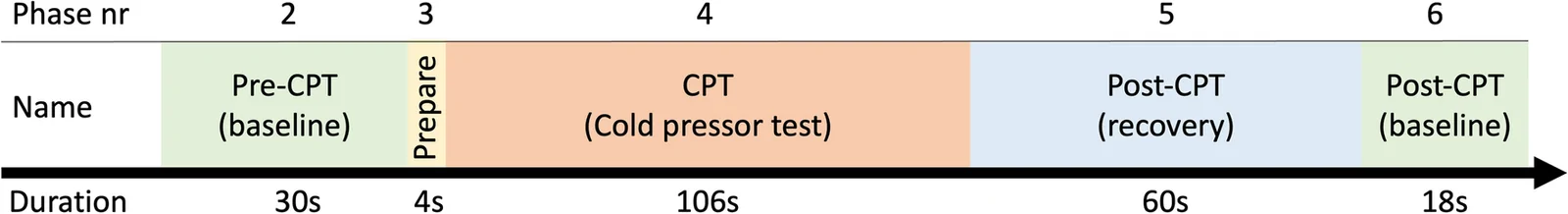
The phases of the cold pressure test (CPT) and approximate duration of each phase. During the CPT (phase 4), participants were free to withdraw their hand from the ice-bath at any time. The maximum duration was 106 seconds. s seconds.

This study uses data from a CPT in the sixth survey of the Tromsø Study (Tromsø6) that was conducted to evaluate pain sensitivity and cardiovascular reactivity. The test is a painful standardized task where participants submerge their hand in cold water. The CPT induces a sympathetic activation and a significant increase in BP. For an overview of the phases of the CPT that are relevant to this paper see Fig. 1, for the complete procedure, please see ref^24^.

During the CPT the participants were continuously monitored with a non-invasive beat-to-beat BP monitor (Finometer Pro; Finapres Medical Systems, Amsterdam, The Netherlands). The Finometer continuously measures BP using the finger clamp method (employing PPG) and provides parameters such as systolic, diastolic and mean arterial pressure, HR, interbeat interval (IBI) and cardiac output^25^. In addition, measures of pulse rate variability (PRV) and BRS was derived from the beat-by-beat measurements. PRV was in this study used as an estimate of HRV, which by standard is calculated from the electrocardiogram (ECG).

In our previous study^26^ we found a significant association between selected PRV measures and the metabolic syndrome, a disorder characterized also by the increased risk of developing CVD. This warranted an investigation on whether the results observed on the group level would translate into a meaningful predictive relationship of future CVD on the individual level.

**Fig. 2:**
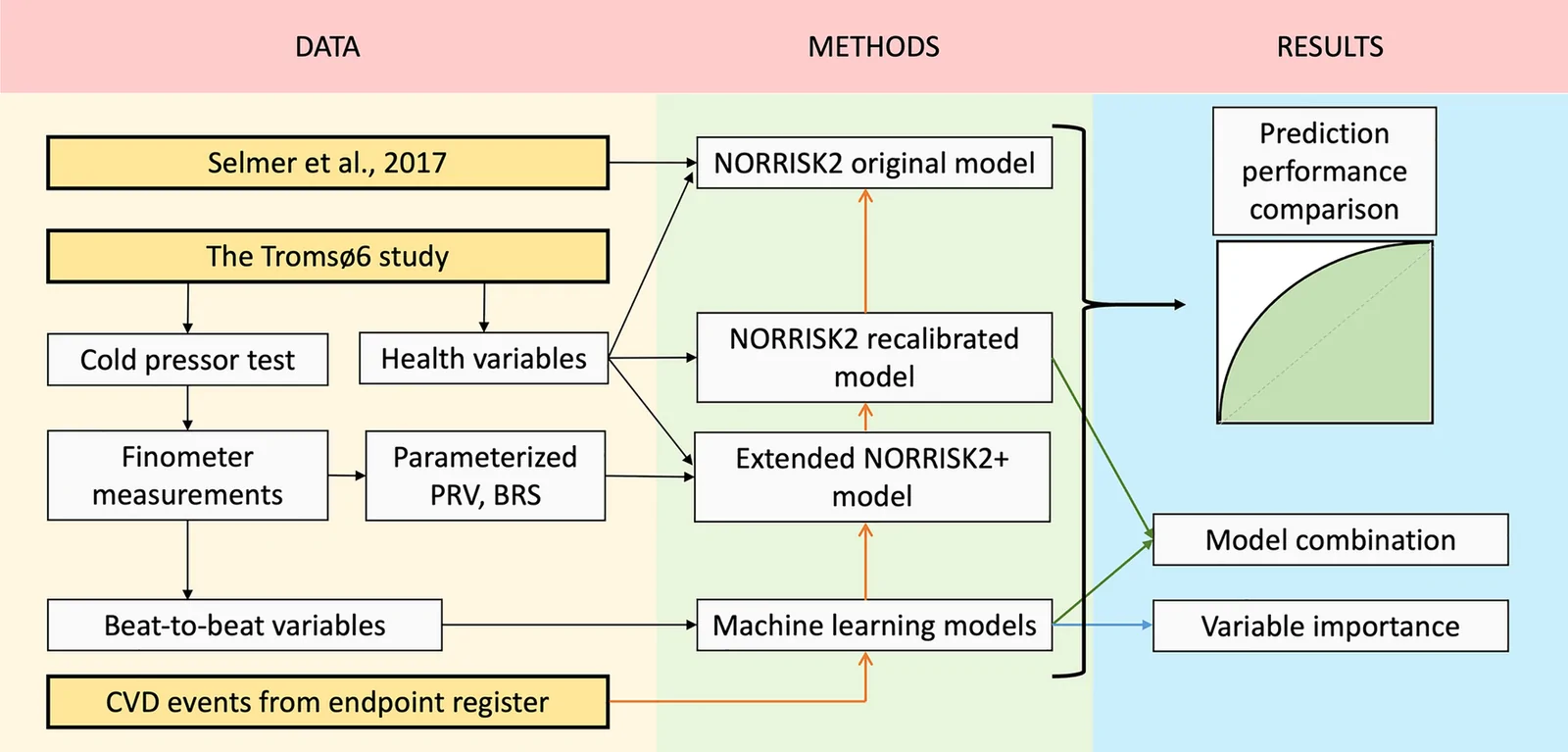
Overview of the workflow: two approaches for improving CVD prediction by using hemodynamic variables were implemented, resulting in the extended NORRISK2 + model and three machine learning models. The original and recalibrated NORRISK2 models were used as baselines for comparisons. The recalibrated NORRISK2 and the best machine learning model were combined with linear regression to investigate if this would improve performance. PRV pulse rate variability, BRS baroreflex sensitivity, CVD cardiovascular disease. The Tromsø Study 2007–8.

With all the variables available in the dataset from Tromsø6 combined with endpoints from the Tromsø Study Cardiovascular Disease Register and the Norwegian Cause of Death Registry, we aimed to investigate the potential for improvement of future CVD prediction by these hemodynamic variables. The workflow of the study is illustrated in Fig. 2. Due to the availability of both single values of resting variables (PRV and BRS) and beat-to-beat time series from the whole CPT, we wanted to compare two risk modeling approaches: One statistical modeling approach based on NORRISK2 with the addition of PRV and BRS (extended NORRISK2 +), and an ML approach that uses the CPT time series without background variables to try to learn patterns in the hemodynamic changes with predictive value for CVD. We investigated if combining the predictions from the recalibrated NORRISK2 model and the ML model in a logistic regression could improve the predictive power of either model. We also hypothesized that the ML model and the original NORRISK2 model would find different subsets of future CVD cases.

## Results

Of the 2495 men in the model development dataset, 144 (5.8%) had a cardiovascular event during the follow-up period. There were 2858 women in the development set, of which 74 (2.6%) had an event. In the model test dataset with 1341 participants, there were 35 men (5.6%) and 19 women (2.7%) with events. Overall (development and test set), the median follow-up duration was 6.6 years; the corresponding medians were 3.5, 4.3 and 6.6 years, respectively, for participants who experienced a CVD event (n = 272), died of other causes (competing risk, n = 178) and did not have an event (censoring, n = 6244).

For the statistical models, the areas under the receiver operating characteristic (AUROC) curve in the test set were 0.8 (95% CI: 0.71–0.86) for the original NORRISK2 model, 0.79 (0.71–0.85) for the recalibrated NORRISK2 and 0.77 (0.69–0.84) for the extended NORRISK2 + model. For the ML models, the AUROCs in the test set were 0.73 (0.67–0.80), 0.70 (0.63–0.76) and 0.62 (0.55–0.70) for the 10-, 4- and 1- variable models respectively (see Table 4 for the specific variables). The combined model had an AUROC of 0.78 (0.72–0.84). The models for women had higher AUROCs than the models for men. Receiver operating characteristic curves and precision-recall curves from the test set are shown in Supplementary Fig. S1.

When comparing the area under the precision-recall curve (AUPRC), the original and recalibrated NORRISK2 models and the combined model had the best result, all with an AUPRC of 0.13. The model with the lowest AUPRC was the 1-variable ML model at 0.07. For AUPRC the results are opposite with respect to the sexes, meaning that the models for the men consistently have a higher value.

**Fig. 3:**
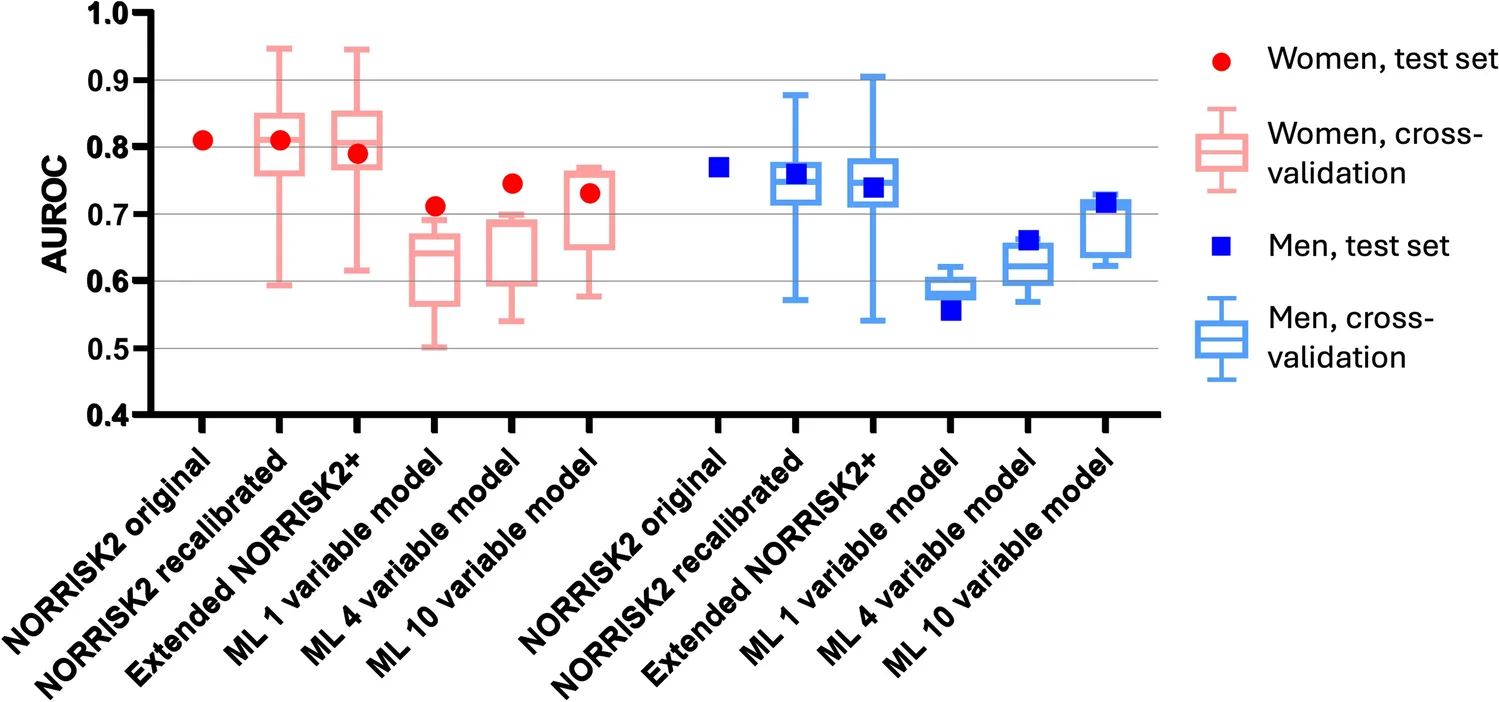
AUROC results from the test set (dots) with the distribution of AUROCs from cross-validation in the development set (boxplots). ML machine learning, AUROC area under the receiver operating characteristic. The Tromsø Study 2007–8.

The AUROCs and AUPRCs (overall and for each sex) from the test set can be found in Table 1. The AUROCs from the test set and the distribution of AUROCs from cross-validation in the development set are shown in Fig. 3. Sensitivity, precision, specificity, accuracy and F1 score can be found in Supplementary Table S1. Confusion matrices from all models can be found in Supplementary Tables S2-S8.

**Table 1:** Evaluation of classification performance on the test set achieved by the different models, on the sample as a whole and separately for each sex.

Model	AUROC			AUPRC		
	Overall	Women	Men	Overall	Women	Men
ML 1 variable	0.62	0.71	0.57	0.06	0.07	0.07
ML 4 variables	0.70	0.75	0.66	0.07	0.06	0.09
ML 10 variables	0.73	0.73	0.72	0.09	0.05	0.12
Original NORRISK2	0.80	0.81	0.77	0.13	0.08	0.16
Recalibrated NORRISK2	0.79	0.82	0.76	0.13	0.09	0.16
Extended NORRISK2 +	0.77	0.79	0.74	0.11	0.09	0.12
Combined	0.78	0.79	0.76	0.13	0.08	0.16

ML machine learning, AUROC area under the receiver operating characteristic, AUPRC area under the precision-recall curve. The Tromsø Study 2007–8.

In addition to comparing the models in terms of AUROC and AUPRC, we were also interested to see to what extent the models found the same individual events. When using a detection threshold corresponding to a sensitivity of 0.5 (meaning that both models would detect 50% of the events), we found that 74% (20 cases) of the true positives were the same in both models, while 26% (7 cases) were unique to each model. Overall, among all predictions, 83% were equal.

The original NORRISK2 model had an AUROC of 0.80 (95% CI: 0.78–0.83) overall, 0.77 (0.73–0.80) for men and 0.83 (0.80–0.86) for women when used to predict on the whole dataset (n = 11 483). The corresponding AUROCs for the recalibrated NORRISK2 model (also on the whole dataset) was 0.81 (95% CI: 0.79–0.83) overall, 0.77 (0.74–0.80) for men and 0.84 (0.80–0.87) for women.

### Global feature importance

**Fig. 4:**
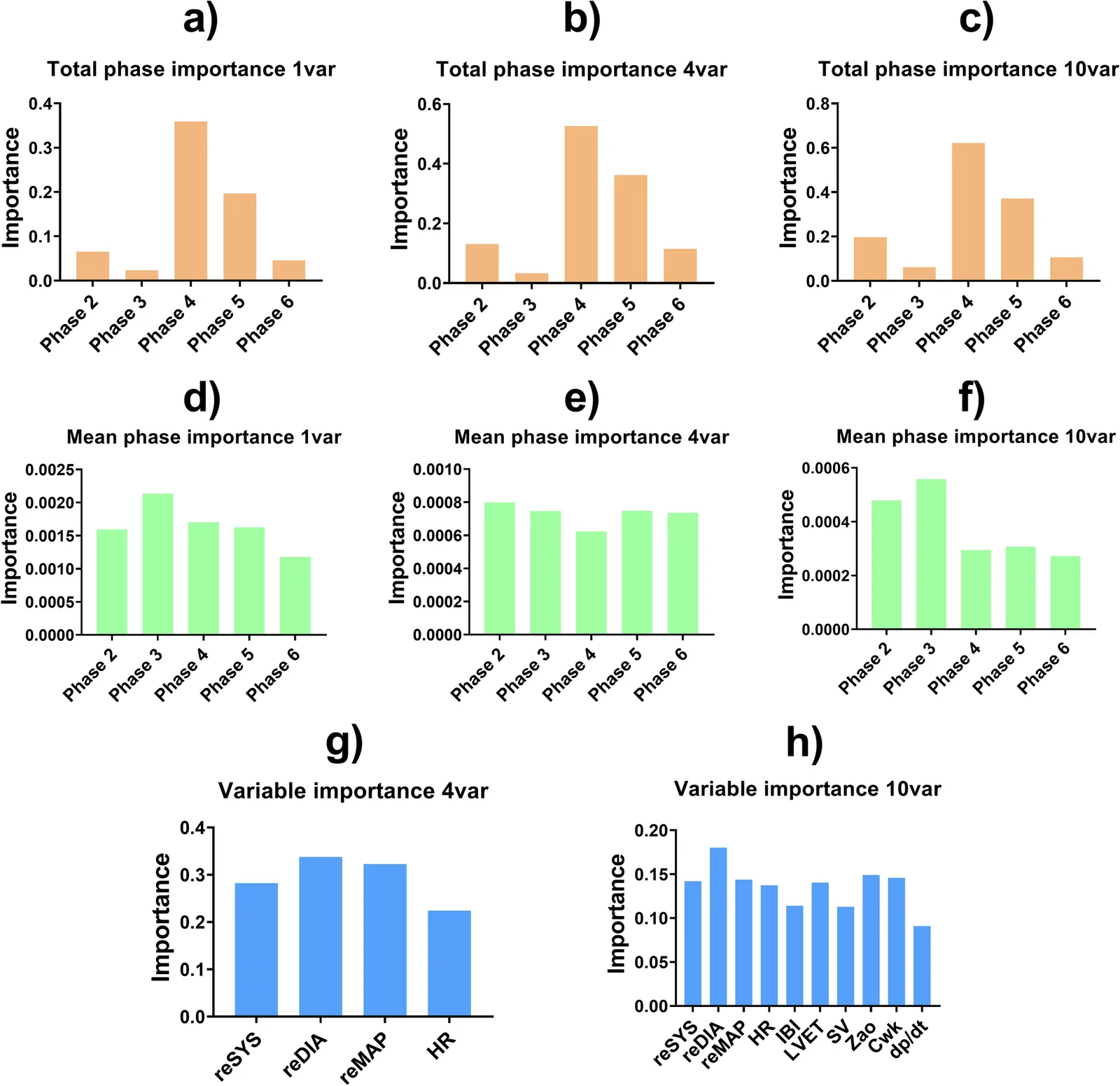
Panels **a**) to **c**) show which phases contribute most to the 1-, 4-, and 10-variable machine learning models respectively. Panels **d**) to **f**) show the same, but here the importance is adjusted for phase length. Panels **g**) and **h**) show relative variable importance for the 4- and 10-variable models. The variables are explained in Table 4. The phases in chronological order: pre-CPT baseline (phase 2), CPT preparation (phase 3), CPT (phase 4), post-CPT period (phase 5), and another baseline period (phase 6). CPT cold pressure test, var variable. The Tromsø Study 2007–8.

We evaluated the global CPT phase importance and variable importance by applying random permutations to the input data and observe the variable’s influence on the final prediction. The results showed that of the phases (see Fig. 1 for an overview), the CPT phase was the most influential in terms of total phase importance for both the 1-variable (Fig. 4 a), 4-variable (Fig. 4 b) and 10-variable (Fig.4 c) models. When the phase importances were scaled according to phase length, the preparation phase was more influential for the 1-variable (Fig. 4 d) and 10-variable (Fig. 4 f) models, while the pre-CPT phase showed slightly higher importance for the 4-variable model (Fig. 4 e). Diastolic blood pressure (DBP) showed highest importance for both the 4-variable model (Fig. 4 g) and the 10-variable model (Fig. 4 h).

## Discussion

### Summary of results

In this study, we sought to find out whether the Tromsø6 PRV and BRS variables had predictive value at the individual level when added to the NORRISK2 prediction model for future CVD. In addition, we tested the CVD predictive ability of only the raw beat-to-beat hemodynamic time series from the same Tromsø6 sample using ML.

The three statistical models (original, recalibrated and extended NORRISK2) performed similarly in the test set in terms of AUROC, while the ML models had lower AUROCs. More variables included in the ML model gave better performance. All models had a higher AUROC for women than for men, as was also the case in the original NORRISK2 publication. However, for AUPRC as a performance metric, the models scored consistently higher in males. Combining the recalibrated NORRISK2 model and the ML model with 10 variables in a logistic regression did not improve the results. When comparing the 10-variable ML model with the extended NORRISK2 + predictions using a fixed detection threshold, 74% (20 cases) of the true positives were the same in both models. Overall, 83% of the predictions were equal. Explainable AI showed that the CPT period, which was the longest, was the most important period for predicting outcomes. In both the 4- and 10-variable models, DBP had the greatest feature importance, though this was most pronounced in the 10-variable model.

### The statistical models

The AUROC from the original NORRISK2 modelling population was 0.84 for women and 0.79 for men, and “slightly lower” in the external validation population^5^. The highest AUROC in our test set came from applying the NORRISK2 model coefficients to our data (the original model), which gave a slightly lower AUROC in our data than on the original dataset. This is to be expected, as Selmer et al.’s numbers were from the original modelling population and from their development set, both factors in favor of higher performance measures. It was more unexpected that the original model performed better or as good as the recalibrated model, although they had a similar performance considering the range of uncertainty. This might be because a part of NORRISK2’s development dataset was from Tromsø4, meaning that the population is very similar to, and to some extent overlapping with, the Tromsø6 population used in this study. In addition, Selmer et al. had a much larger development sample size, meaning that the model parameters could be estimated more accurately.

The model populations differed in that NORRISK2 only included participants between the age of 40–79 while this study did not filter on age, resulting in an age range of 30–87, though only 377 participants were outside the age range of NORRISK2.

Although we see a slight reduction in AUROC when adding the PRV and BRS variables, this is within the margin of error (the confidence interval of the recalibrated model covers the estimate for the extended model) and thus not a significant difference. This result shows that the inclusion of our PRV and BRS variables in the NORRISK2 model did not enhance its predictive performance.

However, we observed a bivariate association between many of the PRV and BRS variables and CVD, indicating that the hemodynamic variables themselves have a relationship with CVD when considered individually (see Supplementary Tables S9 and S10). There are several potential reasons why including them may not improve the multivariate model even if there is a true bivariate effect, one being a weak association to the outcome combined with insufficient statistical power to detect it, another could be multicollinearity with the conventional variables. However, the multicollinearity was found to be low, suggesting that this is not the cause. Additionally, the bivariate association might not reflect a genuine relationship, e.g. due to confounding factors or random noise. Adding variables to a model increases its complexity and lowers the ratio of observations to parameters, leaving the model with fewer degrees of freedom for error estimation. If the added variables do not contribute sufficient unique predictive information beyond the existing variables (redundancy), their inclusion is unlikely to improve, and may slightly reduce, the predictive performance, consistent with what we observed.

A well-known rule of thumb in survival modeling suggests having at least 10 events per candidate predictor parameter (β-term) estimated in the modeling process^27,28^ (i.e. including those that are only considered but not included in the final model^29^). We estimated 22 candidate predictor parameters for the male model and 21 for the female model, meaning that if we were to fulfill the rule of thumb, we would need 210–220 cardiovascular events in the development data alone. The number of events in men was 144, and only 74 in women.

Despite the lower number of events available for training among female participants, all models obtained a larger AUROC for them, including the ML models that were not trained separately for the sexes. This is likely an effect of women having a lower incidence of CVD than men, making their dataset even more imbalanced. The ROC curve uses the false positive rate (1 minus specificity) on one axis, which is not particularly sensitive to the number of false positives when there are many negatives in the dataset^30^. This is, however, a highly relevant number when there are few true positives. The PRC uses precision (positive predictive value) instead, which is much more sensitive to the number of false positives in imbalanced datasets. The AUPRC is therefore known to be a better suited performance measure when the classes are very imbalanced^31^. As shown in Table 1, the AUPRC is higher for men in all the models tested, which supports this interpretation.

To get a more nuanced and concrete picture than what the AUROCs and AUPRCs alone can give, we can make an example based on the measures in Supplementary Table S1. If we have 10 000 patients and a known CVD incidence of 4%, a sensitivity of 0.43 (as in the original NORRISK2 model with a threshold selected to maximize the F1 score) would translate into 172 of the 400 future cases being detected. The corresponding specificity of 0.91 would give 864 false positives. Furthermore, the results for the extended model would be 224 of 400 cases detected (sensitivity of 0.56), and 1536 false positives (specificity of 0.84). These examples illustrate the challenges of predicting an endpoint with a low incidence.

### Machine learning models

The ML models had lower AUROCs and AUPRCs than the statistical models, yet still with a significant ability to predict the outcome without the use of conventional variables, as in NORRISK2. This is to be expected of the 4- and 10-variable models, as the hemodynamic variables are derived from the noninvasive BP waveform, and the predictive value of BP on CVD is well established. It is interesting to note that the model which included only IBI (1-variable model) also had significant predictive power, although with accuracy likely below clinical interest. The ML model with the most variables achieved the strongest predictive score, meaning that the combination of different hemodynamic variables improved the prediction. This also suggests that an ML approach to learn features from the raw BP waveform may reveal even better models without the possible limitation imposed by the selection of predefined variables derived from the BP waveform.

Combining the predicted probabilities from the 10-variable ML model and the recalibrated NORRISK2 model in a logistic regression did not improve the performance. The coefficient for the NORRISK2 score was much larger than that for the ML score, meaning that the NORRISK2 model had a higher influence on the combined model’s predictions. In agreement with this, the overlap in predictions between the 10-variable ML model and the extended NORRISK2 + model was quite large. This indicates that the information with predictive value (that the ML model picks up from the 10 chosen variables) overlaps with the information in the NORRISK2 variables.

The ML models presented here rest on a different mathematical framework than the statistical models. First, the ML input data are different, being time-series beat-to-beat data also including the CPT phase, where we chose not to include background variables about the participants. In this way, we test the predictive value of the information in the hemodynamic time-series data exclusively, unaffected by the conventional variables. Secondly, we did not model the ML models separately for women and men. In the case of the statistical models, it was natural to model the sexes separately to match the NORRISK2 framework, and since the traditional variables are known to exhibit sex differences. In contrast, we wanted to impose as few constraints on the ML models as possible, to allow for new patterns to be learned from a type of data where prior knowledge of sex differences is limited. In addition, ML models in general need more data to be able to make good predictions and stratifying on sex could make both models weaker.

The 1-variable ML model uses only IBI time series to predict CVD, and this approach achieves the poorest result among the models in this study. However, this is of interest considering that IBI is one of the most accessible variables included in the study, available in consumer wearable devices with PPG or ECG measurement. In contrast, the 4- and 10-variable models performed better, but with respect to availability of technology and measurements, they require other sensors that are not yet widely implemented in wearables (see details on cuffed and cuffless solutions in the introduction). This means that implementing one of those models would currently require sophisticated measurements until reliable wearable BP measurement is available^32^. On the other hand, the NORRISK2 score requires an examination and blood test, and the performance of the 1-variable ML model depends on the CPT period, which is also not commonly available even if a person has a smart watch.

If the hemodynamic sensor technology and ML models were improved, one could imagine that the NORRISK2 model could function as a screening process, filtering out people at higher risk that could then get a second check including hemodynamics analyzed with an ML model to confirm the risk. Instead of the CPT, it would be interesting to see if the results would change with a test designed for checking cardiovascular health. Tests of cardiovascular response, such as the Valsalva maneuver, controlled deep breaths, or use of a hand dynamometer, are forms of provocations that trigger the nervous system and can be expected to elicit a response in the patient’s hemodynamics^33^. Another benefit of these types of tests is that they can be conducted more feasibly in a doctor’s office than the CPT, which requires a bucket of ice water, and induces severe pain in most patients.

### Variable importance

The importance of the test phases (assessed by the permutation feature importance approach) indicated that the CPT phase (phase 4) dominated in terms of total contribution to the prediction (Fig. 4 a-c). However, these phases are of different lengths, and calculating the mean phase importance within each phase yields a more even importance (per time unit) across the phases (Fig. 4 d-f). The distribution in importance between the phases was comparable between the 1-, 4- and 10-variable models with both approaches.

The CPT phase was not equally long for all recordings, as participants could withdraw their hand before the end of the test, and shorter recordings were zero-padded. We do not know if individual pain endurance time may therefore be part of the predictive information utilized by the model or how it affected the importance of the CPT phase. It is also possible that the duration of the test affects the hemodynamic response, resulting in pain sensitivity implicitly driving results. Even though pain tolerance has been shown to be related to various CVD outcomes, including in the Tromsø6 data^34^, the test duration was not found to be a significant explanatory variable for CVD in a survival model in this sample.

The variable importance (Fig. 4 g-h) showed that DBP presented slightly higher importance than the other hemodynamic variables in both the 4- and 10-variable models, although other variables were of comparable importance. Because the differences are relatively small and the method does not produce estimates of the uncertainty, these findings should be interpreted with caution. We cannot conclude that DBP is the main causal determinant of long-term CVD risk in this context, given that systolic BP (SBP), HR, and IBI are physiologically and statistically correlated with DBP and with each other. When multiple predictors have overlapping information, permutation-based (or related) importance measures may overestimate the predictive value of one variable, while underestimating the contribution of others, which is a well-known consequence of multicollinearity.

Nonetheless, it is also plausible that DBP provides a more direct representation of vascular resistance and arterial tone and therefore may be more strongly linked to long-term CVD risk. In contrast, SBP, HR, and IBI are more likely to reflect acute cardiovascular reactivity to stress, such as the CPT, which may have a different predictive relevance for long-term outcomes. The variable importance results shown here are compatible with the literature, which shows that DBP and SBP have similar importance in risk prediction of vascular mortality, though SBP is found to be slightly more informative^35^.

### Limitations

Several limitations of this study should be acknowledged. As discussed above, the limiting factor for survival models is not the number of participants, but rather the number of participants with the event of interest. Therefore, even though the Tromsø6 dataset includes many participants, the statistical power after dividing the dataset into development and test sets (and separating the sexes for the statistical models) may have been too low to estimate the parameters adequately. This concern is amplified by the fact that the recalibration did not improve upon the original NORRISK2 coefficients. This affects how confident we can be when comparing model performances and drawing conclusions about the usefulness of the hemodynamic data.

Even though the ML modelling involved nine different candidate models during model development, including different loss functions and hyperparameter space, this search for performance optimization was far from exhaustive and limited by computational resources. This also includes the selection of hemodynamic variables from the Finometer and the preprocessing methods, suggesting that further optimization is possible using this or other datasets.

Another limitation is that the derived PRV variables used in the statistical models are based on ultra-short (30 or 60 s) plethysmographic measurements intended as a surrogate for HRV that is conventionally based on longer ECG recordings, and are likely less accurate than standard HRV measurements. Although these variables have earlier shown statistically significant groupwise differences^26^, a large within-group variation was also observed. The variables’ inaccuracies may reduce their ability to provide predictive information on an individual level.

Regarding PRV vs HRV, Yuda et al.^36^ argued that PRV should not be seen as a surrogate for HRV, but rather a different biomarker in its own right, and they encouraged studies into the biomedical usefulness of PRV independent of HRV. They concluded that even though “HRV is a major source for PRV, PRV is generated and modulated by many other sources and factors, which could contain useful biomedical information that is neither error nor noise”. In light of this perspective, and the availability of PRV compared to HRV, it might be a more relevant goal to find circumstances where PRV has predictive value, rather than focusing on the agreement between HRV and PRV.

Participants with atrial fibrillation were not excluded from the analyses in this study to maintain consistency with the original NORRISK2 study population, which included these individuals. This might have introduced some noise, as these patients presumably both have a higher PRV and a higher probability of developing other CVDs, which is the opposite association compared to the typical direction seen in healthy subjects. There were 193 participants included in the development set with self-reported atrial fibrillation. A sensitivity analysis was done with the original NORRISK2 coefficients, which showed no difference in results, except slightly wider confidence intervals. The stability of the results suggests that excluding the 193 participants with atrial fibrillation would not reduce the overall variability in the results substantially.

### Future perspectives

This study used beat-to-beat hemodynamic time series as input to train ML models. It is also possible that raw sensor signals (such as the BP waveform) with high time resolution may provide more predictive information that can be learned through suitable ML approaches. We consider this to be relevant work for a future study on the possibilities of using hemodynamic waveform data in CVD prediction, also backed up by results from recent studies^37–39^.

The analysis of global feature importance showed DBP as the most influential variable and the CPT phase as the most important period. In future work one could expand upon these results by exploring conditional permutation importance or other methods that are able to compensate for multicollinearity during feature importance calculation^40^.

Given that DBP was identified as the most influential variable, it would be interesting to build a 1-variable ML model with only DBP as a predictor and compare it to the 4- and 10-variable ML models from this study to see how much of their predictive ability comes from this variable. It could also be explored if the variables that are highlighted as having the biggest feature importance can function as predictors independent of their time-series nature. For example, by comparing prediction models using the variables as time series to models using only the mean values of the same variables. If mean values (or other types of summary measures, such as the maximum change due to the CPT) perform comparably to the time series, it would be preferable to use them since time-series data require more advanced models.

In this study, one of the aims was to test the independent predictive power of the hemodynamic time-series data in an ML model without the use of conventional variables. A natural next step would be to develop a multimodal ML model combining both time-series data and conventional tabular variables. A pragmatic initial approach would be to add the conventional variables to the end of the hemodynamic time series and apply the existing Bagging&Boosting model. This strategy is feasible because ensemble methods are generally well-suited for tabular data, though they are often less effective for raw physiological signals. However, the high performance of the Bagging & Boosting model (compared to more frequently used signal interpretation models such as convolutional and recurrent neural networks) observed in the present work is likely due to the highly standardized measurement protocol, which enables the model to align and compare corresponding samples across subjects. This consistency reduces the need for explicit modeling of temporal slopes or complex signal patterns, thereby allowing ensemble methods to extract predictive information effectively from the hemodynamic data presented in this study.

Alternatively, another promising approach would be to first train a convolutional neural network-based autoencoder to reconstruct its own input signal, thereby learning a compact and informative latent representation of the data in a self-supervised manner. The encoder part of the model can then be used to extract these compressed latent features from the test data. These compressed representations can subsequently be combined with the standard NORRISK2 features to form an enriched feature set.

We cannot rule out the possibility that with more CVD cases or more accurate PRV and BRS measurements, the results would have been more positive with respect to prediction improvement by addition of these variables. It is possible to do a more fine-tuned power analysis than to rely on the 10 events per variable rule of thumb^29^. With a study that uses a test specifically designed to assess cardiovascular health, has a larger number of participants with CVD and perhaps with better quality of PRV and BRS variables, one could validate or improve upon the findings of this study.

## Conclusion

Even though we saw a bivariate association between PRV and BRS variables and CVD, adding the chosen summary measures from the Tromsø6 dataset did not improve upon the established NORRISK2 model’s predictive ability.

There is relevant information in the beat-to-beat time series of hemodynamic variables from a CPT that has a significant (p < 0.01) ability to predict future CVD without any other person-specific data. It seems this information has a big overlap with the information in the traditional risk factors for CVD, as the models found many of the same participants at risk, and because there did not seem to be any gain in prediction performance when the ML model was combined with the traditional NORRISK2 (recalibrated version), even though the ML model itself had a statistically significant predictive capability. Although adding these hemodynamic variables did not reveal CVD prediction improvement for this dataset, we do not exclude the possibility of model improvement employing larger datasets or more accurate measurements, or by also using the complete pulse waveform data in model development.

## Methods

### Data source

The data used in this study is from the sixth survey of the Tromsø study in 2007–2008. The Tromsø study is a prospective population study that had been carried out in the north of Norway since 1974. Tromsø6 included questionnaires, interviews, anthropometric measurements, clinical examinations, sampling of biological materials and a CPT. Sampling procedures can be found in^41^. For more detailed information about the data relevant to this paper, see^26^.

The Tromsø study maintained a complete cardiovascular endpoint register called the Tromsø Study Cardiovascular Disease Register, until the end of 2014. The register included data from the Norwegian Cause of Death Registry such that it contained both verified CVD diagnoses and the date of death from all causes. The endpoint register was used to define the study endpoints and, in combination with the questionnaires from Tromsø6, to determine the participants’ history of CVD.

The Norwegian Data Protection Authority and the Regional Committee of Medical and Health Research Ethics, North Norway have approved the Tromsø6 study. The study complies with the Declaration of Helsinki, and each participant gave written informed consent prior to participation. This study is a part of a project called “Heart Rate Variability and Baroreceptor Sensitivity in Relation to Pain and Relevance to Cardiovascular Morbidity and Mortality in a General Population: The Tromsø Study” that was approved by the Regional Committee for Medical and Health Research Ethics, North Norway (2019/143, REK ID 8885).

### Dataset

An overview of the workflow of this paper is shown in Fig. 2 The hemodynamic variables used in this study come from the continuous measurement of BP with a Finometer (Finometer Pro; Finapres Medical Systems, Amsterdam, The Netherlands) during the CPT at Tromsø6.

The CPT had seven phases, of which phases 2–6 are relevant for this study. In chronological order, they are pre-CPT baseline, CPT preparation, CPT, post-CPT period and another baseline period (see Fig. 1). The PRV and BRS measures were calculated from two resting periods: phases 2 (approximately 30 s) and 5 (approximately 60 s). The time-series data used in the ML model are from all the phases 2–6. The CPT period was not used to calculate PRV and BRS measures to avoid the confounding effect of the transient hemodynamic response to dipping the hand in ice-cold water. The participants of the CPT were free to withdraw their hand from the ice-bath at any time.

Of the 9946 hemodynamic recordings, 1660 were excluded based on missing data or phase labels, and 1098 had poor data quality, including 368 records excluded based on the visual inspection of all records (by authors NA and CT, blinded to outcomes and excluded before data analysis), with the intent of removing records with obvious technical errors.

**Fig. 5:**
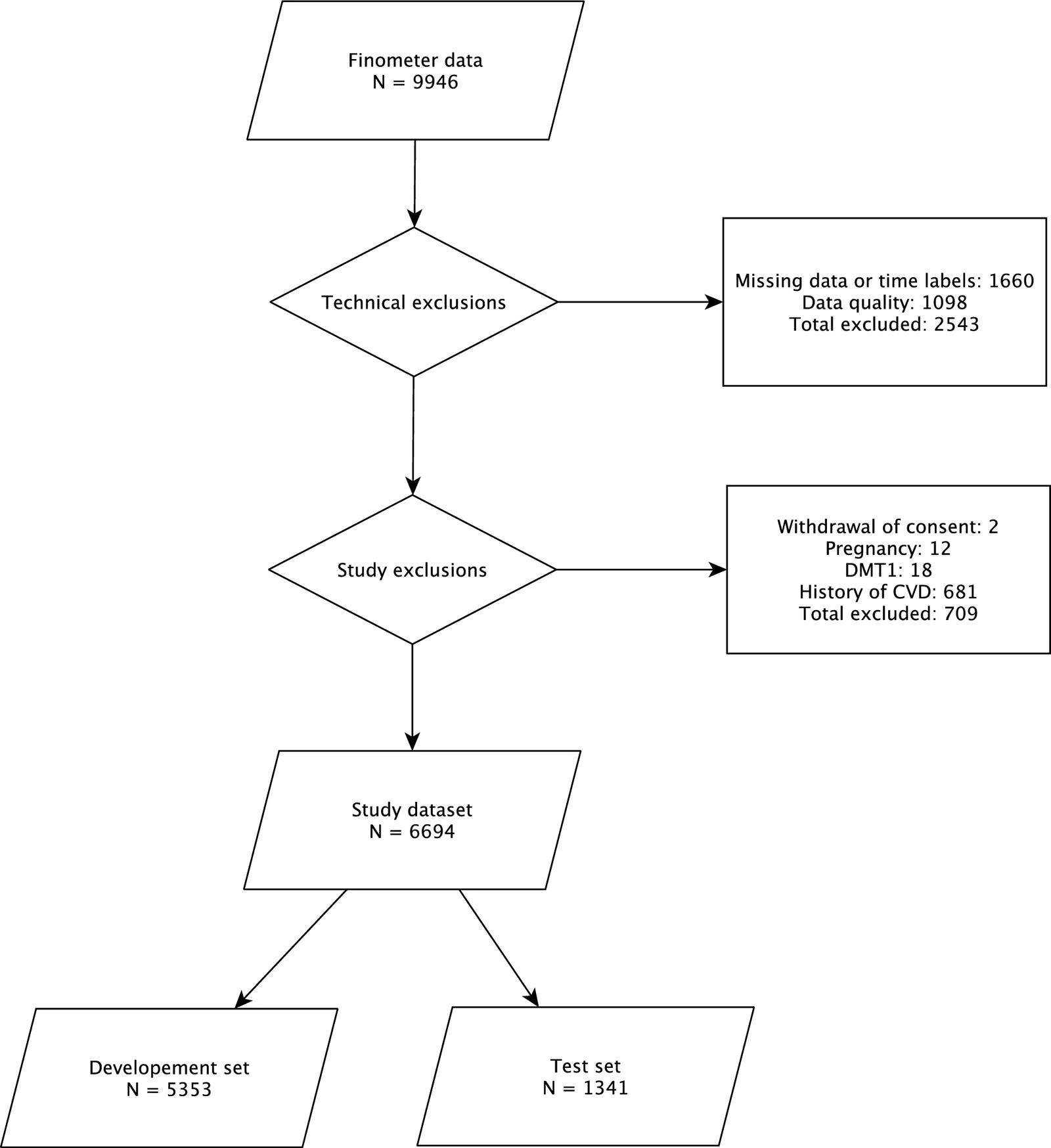
Flowchart describing exclusions leading to the final datasets for developing and testing the machine learning and statistical models. DMT1 diabetes mellitus type 1, CVD cardiovascular disease. The Tromsø Study 2007–8.

The medical exclusions made were of pregnant participants, participants with assumed type 1 diabetes (diagnosed before or at the age of 30 from questionnaire) and participants with a history of CVD (stroke, MI, or angina). To exclude the latter group based on variables that included missing values, we imputed the CVD history. Out of 144 participants with missing values, three were imputed to having had CVD in more than half of the 20 imputed datasets, resulting in a total of 681 participants being excluded for this reason. The process of exclusion is shown in Fig.5, and the population characteristics of the remaining participants are shown in Table 2.

**Table 2:** Characteristics of the 6694 participants in the development and test set.

Variable	Mean (SD) or percentage (n)		% missing
	Men (N = 3120, 46.6%)	Women (N = 3574, 53.4%)	
Age year (SD)	55.5 (11.5)	56.2 (11.8)	0.0
Systolic blood pressure mmHg (SD)	136.8 (19.3)	132.1 (23.5)	0.3
Serum total cholesterol mmol/l (SD)	5.6 (1.0)	5.8 (1.1)	0.9
Daily smoking % (n)	19.2 (593)	21.5 (757)	1.2
Antihypertensive medication % (n)	17.1 (530)	18.2 (645)	0.7
Low HDL-cholesterol % (n)	10.7 (331)	17.3 (612)	0.9
Family history of CVD: one member % (n)	15.3 (477)	18.9 (675)	*
Family history of CVD: two members % (n)	1.0 (30)	1.8 (65)	*
Future heart attack % (n)	3.6 (111)	1.2 (42)	*
Future stroke % (n)	2.4 (75)	1.5 (54)	*
SDNN pre-CPT (SD)	41.1 (24.9)	37.9 (21.5)	0
SDNN post CPT (SD)	57.5 (32.5)	55.6 (29.6)	0
RMSSD post CPT (SD)	34.9 (27.7)	34.9 (23.2)	0
PNN50 pre-CPT (SD)	11.9 (15.8)	12.4 (15.7)	0
PNN50 post CPT (SD)	13.0 (16.3)	14.0 (15.8)	0
WAVD8 HF pre-CPT (SD)	390.4 (885.9)	366.1 (611.0)	0
WAVD8 HF post CPT (SD)	429.3 (923.4)	418.3 (618.5)	0
BRS pre-CPT (SD)	10.8 (15.2)	11.2 (17.6)	38.9
SDNN pre-CPT (SD)	3.6 (0.6)	3.5 (0.5)	0
SDNN post CPT (SD)	3.9 (0.6)	3.9 (0.5)	0
RMSSD post CPT (SD)	3.4 (0.6)	3.4 (0.6)	0
PNN50 pre-CPT (SD)	0.9 (2.4)	1.0 (2.3)	0
PNN50 post CPT (SD)	1.3 (2.1)	1.5 (2.1)	0
WAVD8 HF pre-CPT (SD)	5.2 (1.2)	5.3 (1.1)	0
WAVD8 HF post CPT (SD)	5.3 (1.1)	5.4 (1.1)	0
BRS pre-CPT (SD)	2.0 (0.8)	2.0 (0.8)	38.9

HDL high density lipoprotein, CVD cardiovascular disease, BRS baroreflex sensitivity, SD standard deviation, CPT cold-pressor test. SDNN, RMSSD, PNN50 and WAVD8 are different measures of pulse rate variability (PRV). The PRV and BRS variables were log transformed before mean and SD were calculated. HDL is considered low if it is lower than 1.3 mmol/l for women and 1.0 mmol/l for men. *Missing was used as negative. The Tromsø Study 2007–8.

After all exclusions, 6694 participants were included for analysis and partitioned into an 80% development dataset used for statistical and ML model development and a 20% test dataset that was set aside until the final models were selected for validation. To ensure similar distribution of important variables in both data sets, the partitioning was stratified according to future stroke and MI with the splitstackshape package in R (version 1.4.8).

In this study, CVD was defined as MI, cerebral stroke and angina pectoris. From the endpoint register we included ischemic, hemorrhagic and unclassified strokes, but not subarachnoid hemorrhage. These verified diagnoses were used to define the endpoints in this study. They were also used, in combination with the questionnaires from Tromsø6, to determine the participants’ history of CVD, though type of stroke was not specified in the questionnaire. As information about angina was only available through the questionnaire and not in the endpoint register, it was only used to determine historic CVD, but not future endpoints. The register’s information about date of death from all causes made it possible to model the competing risk of dying from other causes.

The NORRISK2 model includes a variable on the number of first-degree relatives with a history of premature coronary heart disease, defined as having an MI before the age of 60. This information came from the questionnaires in the CONOR health surveys (Cohort of Norway https://www.fhi.no/en/hs/conor/), including Tromsø4 and -5), that had one question for each type of first degree relative (mother, father, sister, brother, and child). In the NORRISK2 model this was re-coded such that the variable could take the values “0”, “1”, and “2 or more”. By the time of the Tromsø6 survey, however, this question was rephrased, so that both parents were coded in one variable, and all siblings in another. This means that if there were participants whose both parents or more than one sibling had a premature MI, this would count as two deaths before, but only one death in our data.

## Model comparisons

The aim of this study was to investigate the potential for improvement of CVD prediction by using hemodynamic measurements, to which we had two approaches: one was to fit a statistical model of the same type as the established NORRISK2 model with the addition of hemodynamic single values (PRV and BRS), and the other was to use the beat-to-beat time-series data in an ML model without any background information on the participants. For each approach, many candidate models were tried out in the development set, but only four models were selected for a final comparison in the test set and presented in the result section. These were the extended NORRISK2 + model and three versions of the same type of ML model, developed on a dataset with 10, 4 and 1 selected hemodynamic variables. In addition to being compared to each other, the models were also compared to the NORRISK2 model as it was recalibrated to our data. The recalibration was done in order to provide a baseline that would enable a fair comparison to the original NORRISK2 model. A logistic regression model that combined the predictions from the 10-variable ML model and the recalibrated NORRISK2 model (averaged over the imputed datasets and min–max normalized) was fitted to explore if the ML model could add predictive power to the NORRISK2 model. Finally, the recalibrated and original NORRISK2 models were compared to each other on the complete dataset. For an overview of the comparisons, see Table 3.

**Table 3:** Overview of model comparisons. ML machine learning. The Tromsø Study 2007–8.

MODEL 1	MODEL 2	TESTED ON
Recalibrated NORRISK2	Extended NORRISK2 +	Test set
Recalibrated NORRISK2	ML (10, 4, 1 variables)	Test set
Extended NORRISK2 +	ML (10, 4, 1 variables)	Test set
Recalibrated NORRISK2	Combined model	Test set
Original NORRISK2	Recalibrated NORRISK2	Complete dataset

The models were compared in terms of AUROC and AUPRC. To see to what extent the NORRISK2 (recalibrated) and ML model (10 variables) predict the same cases, the overlap in predictions was investigated when both models used a detection threshold corresponding to a sensitivity of 0.5. This means that, by definition, both models detected 50% of the events.

The statistical models were modelled separately for each sex, while the ML models used all participants (in the development set) together. Following is a detailed description of how each model was developed.

## Preprocessing

The hemodynamic recordings had previously been corrected for artifacts using the Deegan et al. method^42^. The complete process of data filtering of suspected artifacts and exclusions of suspected low-quality recordings are described in the Supplementary Methods.

### Statistical models

Missing values were multiply imputed with the MICE package in R (version 3.15.0)^43^ with the default imputation methods for continuous and categorical data. The dataset was imputed 30 times following the *impute, then transform* approach^44^, separately for the NORRISK2 and the extended NORRISK2 + models. The missing data was assumed to be missing at random. All results from the statistical methods presented in this paper were averaged over the imputation sets, following Rubins rules^45^ where appropriate, unless otherwise stated. E.g., an AUC was calculated for each imputed data set, and the average was reported.

The original NORRISK2 variables were transformed following the methodology described by Selmer et al. PRV and BRS variables were centered and log transformed.

### Machine learning models

Three datasets were processed for ML training and prediction: One with time series of ten selected Finometer variables, one with only BP (systolic, diastolic and mean arterial pressure) and HR variables, and one dataset with only the interbeat interval time series representing measurements that can be taken with a typical commercial smart-watch. An overview of the variables can be found in Table 4.

**Table 4:** Description of the selected Finometer variables used in the machine learning models.

Finometer variable name	Description	10-variable input	4-variable input	1-variable input
reSYS	‘reconstructed brachial’ systolic blood pressure, the maximum pressure in arterial systole	X	X	
reDIA	‘reconstructed brachial’ diastolic blood pressure, the lowest pressure just before the next upstroke	X	X	
reMAP	‘reconstructed brachial’ mean blood pressure, the true integrated mean between upstrokes	X	X	
HR	Pulse rate, derived from interbeat interval	X	X	
IBI	Interbeat interval, time between two consecutive upstrokes	X		X
LVET	Left Ventricular Ejection Time, time between upstroke and dicrotic notch	X		
SV	Stroke Volume from Modelflow simulation	X		
Zao	Ascending aorta characteristic Impedance at the current diastolic pressure	X		
Cwk	Total arterial compliance at the current diastolic pressure	X		
dp/dt	Maximum slope of unprocessed pressure rise during the upstroke	X		

The Tromsø Study 2007–8.

To align and format all recordings into the same time scale, time series from the different phases were separately resampled by interpolation according to the target duration of the phases (e.g. 60 s for the post-CPT period), using an even sampling rate of 2 Hz. Of the participants included in the final dataset, 27% withdrew their hand early, resulting in recordings that were shorter than the full CPT period of 106 s. In order to ensure equal data length and time alignment, these missing data points were padded when used as ML data input. The first 10 s of the pre-CPT baseline was excluded as the BP seemed to not have reached a stable level until after this point (based on visual inspection of the mean SBP during this phase). This provided a combination of five phases with the following target durations: Phase 2 (pre-CPT baseline): 20 s, phase 3 (CPT preparation): 5 s, phase 4 (CPT): 105 s, phase 5 (post CPT): 60 s, phase 6 (baseline): 19 s.

Three main preprocessing methods were applied to the data prior to fitting the ML models, though not all methods were used for every model. First, min–max scaling was employed to normalize the feature values to a range between 0 and 1. This was done by fitting the scaler on the development set and then applying it to both the development and test sets. Second, missing values in the time-series data were handled by replacing them with the value of 0. This ensured that the models could process the data without errors due to missing values, e.g. resulting from participants withdrawing their hand before the maximum duration of the CPT. Finally, for models that could not process arrays with more than two dimensions, the time-series data with three dimensions were flattened into two dimensions. This step was necessary to ensure compatibility with the models that had limitations on the data input format. The specific application of these preprocessing steps for each model is detailed in Table 5.

**Table 5:** Overview of the preprocessing methods applied to the different machine learning models that were tried out in the developments set.

Model	Preprocessing		
	Min–Max scaling	Remove missing values	Flatten array
Bagging&Boosting	x	x	x
Gaussian Naive Bayes classifier	x	x	x
Logistic regression	x	x	x
Support Vector Machine	x	x	x
Random Forest	x	x	x
Balanced Random Forest	x	x	x
XGBoost	x		x
CNN models		x	
LSTM	x	x	

CNN convolutional neural network, LSTM long short-term memory. The Tromsø Study 2007–8.

## Prediction modeling

### NORRISK2 (original and recalibrated)

The NORRISK2 model was tested on the Tromsø6 dataset both with the original coefficients and with a recalibrated version. For the recalibrated version, we were given the Stata code used by Selmer et al. to reproduce the exact same competing risk model. This model was trained both on the whole dataset for comparison with the original coefficients, and only on the development set for comparison with the extended NORRISK2 + and ML models. Stata version 16.1.

### Extended statistical model

We wanted to extend the NORRISK2 model by adding PRV and BRS variables, however, these measures can be calculated in several ways that highlights different aspects of the variability in heart rate. Four PRV variables were therefore chosen to complement each other: SDNN (highly correlated with slow variations such as parasympathetically-mediated respiratory sinus arrhythmia^46^), RMSSD (reflecting beat-to-beat variability correlated with high-frequency variability), pNN50 (the percentage of adjacent NN intervals that differ from each other by more than 50 ms), and the power of HRV within the high-frequency range based on the Wavelet transformation using the Daubechies 8 wavelet^47^. In addition, we included a measure of the spontaneous BRS reflecting the functional efficiency in maintaining stable BP in response to changing conditions^48^. Each variable was added from both the pre and post CPT periods.

Six different statistical model selection techniques were then tested (on the pooled dataset after imputation) to narrow down the number of possible combinations of the variables to test on the test set. The techniques were backward selection, forward selection (both methods once with exclusion criterion 0.1 and once with 0.2), and backward and forward stepwise selection (exclusion and inclusion criteria 0.2/0.1 and 0.2/0.15 for backwards and forwards respectively). In addition, the full model, meaning all NORRISK2 variables and all the preselected PRV and BRS variables, was fitted and a threshold of 0.05 used to determine significance. For every variable it was then noted how many times it was included in the models from the selection methods (or significant in accordance with the threshold), and the candidate models were chosen based on this. From these results, four candidate models were selected for further testing for both sexes. These are listed in Table 6 (men) and Table 7 (women). The techniques were run once with log transformed HRV and BRS variables and once with untransformed variables. The transformed PRV and BRS variables were chosen over the untransformed versions, as it was clear that they were generally stronger contributors to the models.

**Table 6:** Candidate models for men.

Variable	Model 1	Model 2	Model 3	Model 4
Age	X	X	X	X
Systolic blood pressure			X	X
Cholesterol	X	X	X	X
Smoker	X	X	X	X
Antihypertensives	X	X	X	X
Low HDL cholesterol	X	X	X	X
Family history of CHD	X	X	X	X
Age X age			X	X
Cholesterol X age	X	X	X	X
Systolic blood pressure X age			X	X
Smoker X age	X	X	X	X
SDNN pre-CPT				
SDNN post CPT		X		X
RMSSD pre-CPT				
RMSSD post CPT				
PNN50 pre-CPT		X		X
PNN50 post CPT				
WAVD8 HF pre-CPT		X		X
WAVD8 HF post CPT		X		X
BRS positive slopes pre-CPT				
BRS positive slopes post CPT				

Note that model 3 is the NORRISK2 model. HDL high density lipoprotein, CHD coronary heart disease, BRS baroreflex sensitivity, CPT cold-pressor test. SDNN, RMSSD, PNN50 and WAVD8 are different measures of pulse rate variability (PRV). All PRV and BRS measures were log-transformed. HDL is considered low if it is lower than 1.0 mmol/l for men. The Tromsø Study 2007–8.

**Table 7:** Candidate models for women.

Variable	Model 1	Model 2	Model 3	Model 4
Age	X	X	X	X
Systolic blood pressure			X	X
Cholesterol			X	X
Smoker	X	X	X	X
Antihypertensives	X	X	X	X
Low HDL cholesterol	X	X	X	X
Family history of CHD			X	X
Age^2^	X	X	X	X
Systolic blood pressure X age			X	X
Smoker X age	X	X	X	X
SDNN pre-CPT		X		X
SDNN post CPT				
RMSSD pre-CPT				
RMSSD post CPT		X		X
PNN50 pre-CPT				
PNN50 post CPT		X		X
WAVD8 HF pre-CPT				
WAVD8 HF post CPT				
BRS positive slopes pre-CPT		X		X
BRS positive slopes post CPT				

Note that model 3 is the NORRISK2 model. HDL high density lipoprotein, CHD coronary heart disease, BRS baroreflex sensitivity, CPT cold-pressor test. SDNN, RMSSD, PNN50 and WAVD8 are different measures of pulse rate variability (PRV). All PRV and BRS measures were log-transformed. HDL is considered low if it is lower than 1.3 mmol/l for women. The Tromsø Study 2007–8.

All the potential models were trained on the development set with tenfold cross-validation. The randomization into the 10 folds was done 10 times, resulting in 100 estimates of AUROC for each model. Based on this, model 4 was chosen as the extended model for both sexes. This model was then rerun on the development set as a whole, and the resulting coefficients were used to predict on the test set. Boxplots based on all the 100 AUROCs for the four candidate models can be found in Supplementary Fig. S2 (men) and S3 (women).

Development and analysis of the extended model was done in Stata version 16.1 and R version 4.2.3.

### Machine learning approaches

Using the three processed Finometer time-series development datasets, a wide selection of different ML models was assessed, comparing 13 different models as listed in Table 8. All models were trained to predict future CVD (as a binary variable) and evaluated within the development dataset using fivefold cross-validation. Model selection was based on comparing the cross-validation performance between the models. As the appropriateness of receiver operating characteristics vs precision-recall plots for use in the case of imbalanced datasets (as in this case) has been debated^31^, we chose to include performance metrics for both the area under the curve of the receiver operating characteristics (AUROC) and the precision-recall curve (AUPRC).

**Table 8:** Selected models and their cross-validation performance in the development set presented according to their rank in performance.

Modell	Variation/hyperparameters	Loss function	AUROC (1, 4, 10 variables)			AUPRC (1, 4, 10 variables)		
Bagging&Boosting		Binary cross-entropy	0.64	0.70	0.74	0.06	0.08	0.11
Gaussian naive bayes classifier^49^			0.62	0.56	0.55	0.04	0.03	0.03
Logistic regression		Logarithmic loss	0.62	0.66	0.67	0.06	0.07	0.08
Support vector machine			0.55	0.59	0.65	0.05	0.12	0.07
Random forest			0.56	0.64	0.68	0.05	0.06	0.07
Balanced random forest			0.64	0.67	0.72	0.06	0.07	0.09
XGBoost			0.61	0.63	0.70	0.06	0.06	0.09
CNN /inceptiontime^50^	Reduce LR based on val_loss	Weighted binary Cross-entropy loss	0.58	0.67	0.69	0.06	0.07	0.09
CNN /inceptiontime	Reduce LR based on val_ROC	Double Soft F1 loss	0.51	0.60	0.65	0.05	0.07	0.06
CNN /inceptiontime	Reduce LR based on val_loss	Double Soft F1 loss	0.52	0.63	0.66	0.05	0.06	0.07
LSTM	Reduce LR based on val_loss	Weighted binary Cross-entropy loss	0.46	0.64	0.72	0.02	0.04	0.06
LSTM	Reduce LR based on val_ROC	Double Soft F1 loss	0.48	0.62	0.68	0.02	0.05	0.05
LSTM	Reduce LR based on val_loss	Double Soft F1 loss	0.51	0.61	0.68	0.02	0.03	0.05

AUROC area under the receiver operating characteristic, AUPRC area under the precision-recall curve, CNN convolutional neural network, LR likelihood ratio, LSTM long short-term memory. The Tromsø Study 2007–8.

Accordingly, we selected the “BaggingBoosting” algorithm, which is a type of ensemble method that combines balanced bagging (bootstrap aggregating)^51^ and histogram based gradient boosting techniques^52^. The balanced bagging classifier addresses the class imbalance in our dataset by training multiple base estimators using balanced bootstrap samples. We configured the balanced bagging classifier to use the histogram-based boosting classifier as the base estimator and set the number of estimators to 10. This setup trains 10 histogram-based boosting classifiers with balanced bootstrap samples and aggregates their predictions. The purpose behind this ensemble approach is to enhance the model’s generalization and stability. We then trained three variations of this BaggingBoosting model on the whole development dataset, using the 10, 4 and 1 preselected variables, before employing the resulting models on the separate test dataset to estimate the performance of these models on unseen data from the same population.

Machine learning development and analysis were performed using Python 3.6.3. The implementation of gaussian naive bayes, logistic regression, support vector machine and random forest were done using Scikit-Learn version 0.24.1. The eXtreme Gradient Boosting (XGBoost) model was implemented using dmlc XGboost 1.4.2. Balanced random forest and the balanced bagging classifier was implemented using Scikit-Learn version 0.24.1 and imbalanced-learn version 0.8.0. Tensorflow, version 2.4.1, was used to implement the convolutional and recurrent neural networks.

## Explainable AI

The rapid development of artificial intelligence (AI) over the last decade has been driven primarily by more data availability, parallelization and computing power. Better computational capabilities enable heavier computing and more advanced models. However, this evolvement comes with a cost. The complexity of the models sometimes makes their decisions inaccessible to humans, often referred to as the black box phenomenon. This has led to the development of another subfield within AI, called explainable AI. Explainable AI is often divided into two main categories; global explanations and local explanations. Global explanations are used to explain the model’s performance in general for all input data and local explanations are used to explain the model’s interpretation on a single input data. Furthermore, these two main categories can be further subdivided into model-agnostic and model-specific explanations. Model-specific methods can only be applied to a specific model, whereas model-agnostic methods can be applied to any model. In the current study, we employ a global, model-agnostic explanation method called permutation feature importance.

### Permutation feature importance

Permutation feature importance is a method for determining the importance of individual features in an ML model. It works by finding the relationship between the features and the target variable in the dataset. The procedure involves randomly shuffling the values of a single feature, while keeping all other features unchanged. The change in the model’s prediction score, when this single feature is randomly shuffled, is defined to be the permutation feature importance for that feature^53^. Features that are more important to the model’s predictions will tend to cause larger changes to the prediction score when they are shuffled, compared to less important features. This method provides a global, model-agnostic way of assessing the relative importance of the different features used as input to the model.

In our current study, we performed permutation feature importance analysis on the test dataset. This approach ensures that the importance scores reflect the model’s performance on unseen data, providing a more accurate and unbiased assessment of each feature’s contribution to the model’s predictions. Performing this analysis on the development set could lead to over-optimistic and misleading importance scores due to overfitting, as the model (the black box model) has already seen and learned from the data in the development set.

The permutation feature importance analysis was repeated 10 times, evaluating the feature importance for the models trained on the 1-variable, 4-variable, and 10-variable Finometer datasets. Furthermore, we summarized the permutation feature importance scores in two ways: phase-wise and variable-wise. The phase-wise analysis allowed us to determine which measurement phase had the greatest predictive power to predict CVD. The variable-wise analysis enabled us to identify which Finometer features were most influential for CVD prediction.

## Supplementary Information


Supplementary Information.


## Data Availability

The dataset analyzed in the current study is not publicly available, but access can be requested from the Tromsø study (https://uit.no/research/tromsostudy/project?pid=709148). To respect terms of use and protect human privacy, the authors cannot directly share the dataset.
