# Supplementary material for: Exploring hemodynamic measurements from the Tromsø Study for prediction of cardiovascular disease using traditional statistical models and machine learning approaches
Source: Sci Rep. 2026 Jun 13;16:27012. doi: 10.1038/s41598-026-57003-5 (PMC13521920; doi:10.1038/s41598-026-57003-5)
Supplement: Supplementary file 1 [file 41598_2026_57003_MOESM1_ESM.pdf]

Supplementary Information

Can hemodynamic measurements improve prediction of cardiovascular disease? A study comparing traditional statistical models with machine learning approaches. The Tromsø Study

Naomi Azulay, Bjørn-Jostein Singstad, Henrik Schirmer, Maja-Lisa Løchen, Roy Bjørkholt Olsen, Trond Geir Jenssen, Audun Stubhaug, Christopher Sivert Nielsen, Leiv Arne Rosseland, Christian Tronstad

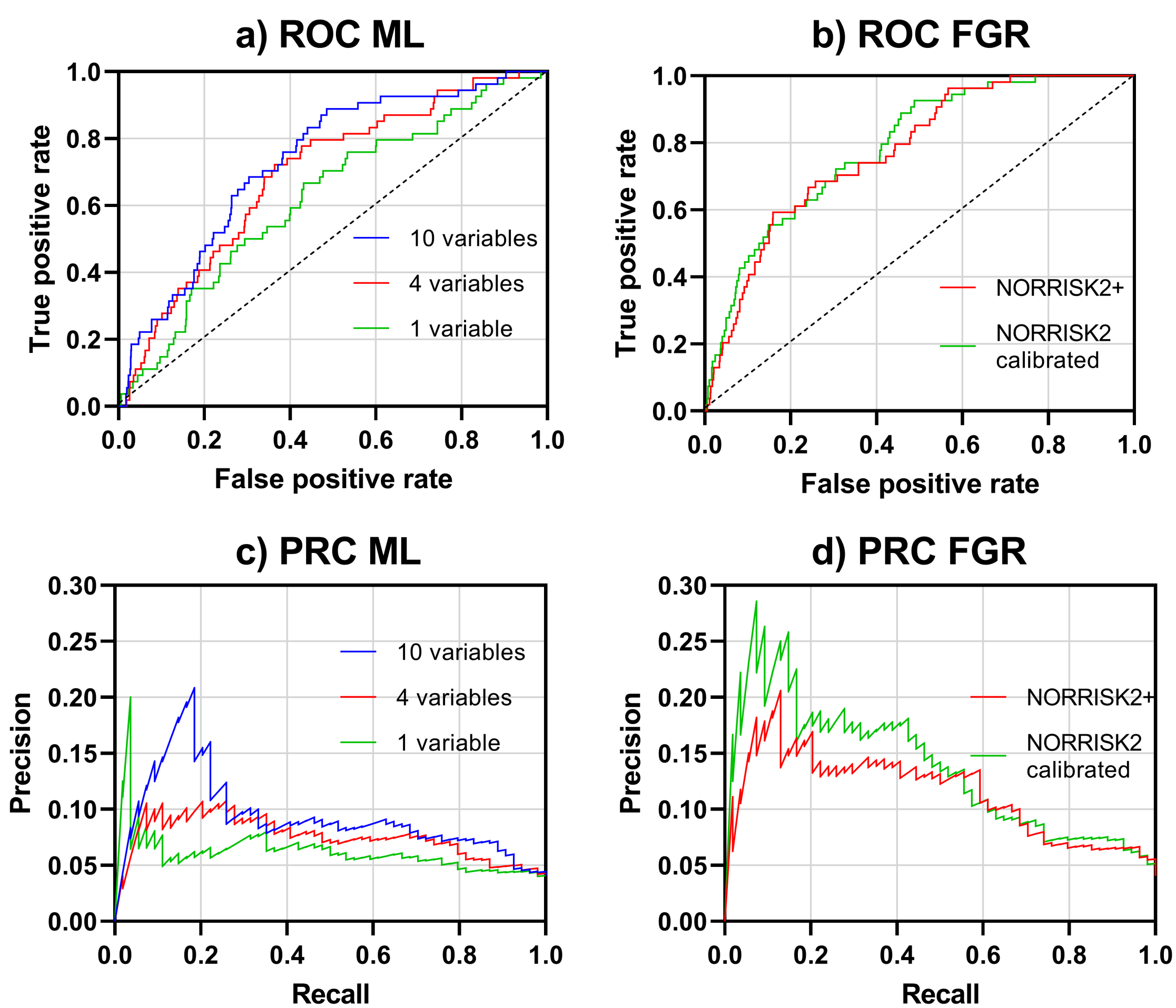

Figure S1. ROC curves and PRC in the test set for the three bagging&boosting ML models and two statistical Fine-Gray models compared in the study. ROC receiver operating characteristic, PRC precision-recall curve, ML machine learning, FGR Fine-Gray regression. The Tromsø study 2007-8.

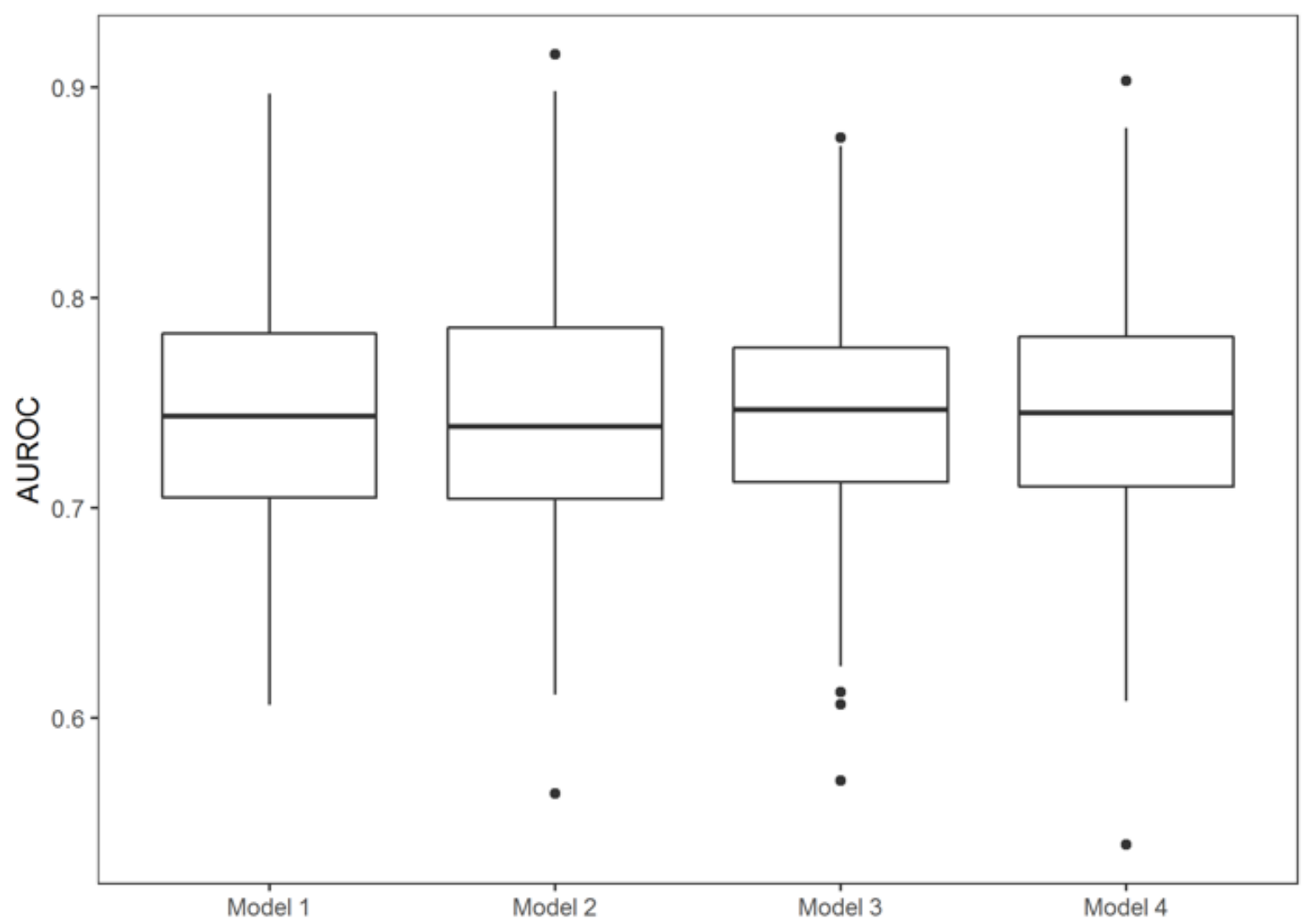

Figure S2. The distribution of AUROC from 10-fold cross-validation in the development set (with ten different randomizations) for the three potential plus-models and NORRISK2 (model 4) for men. AUROC area under the receiver operating characteristic. The Tromsø Study 2007-8.

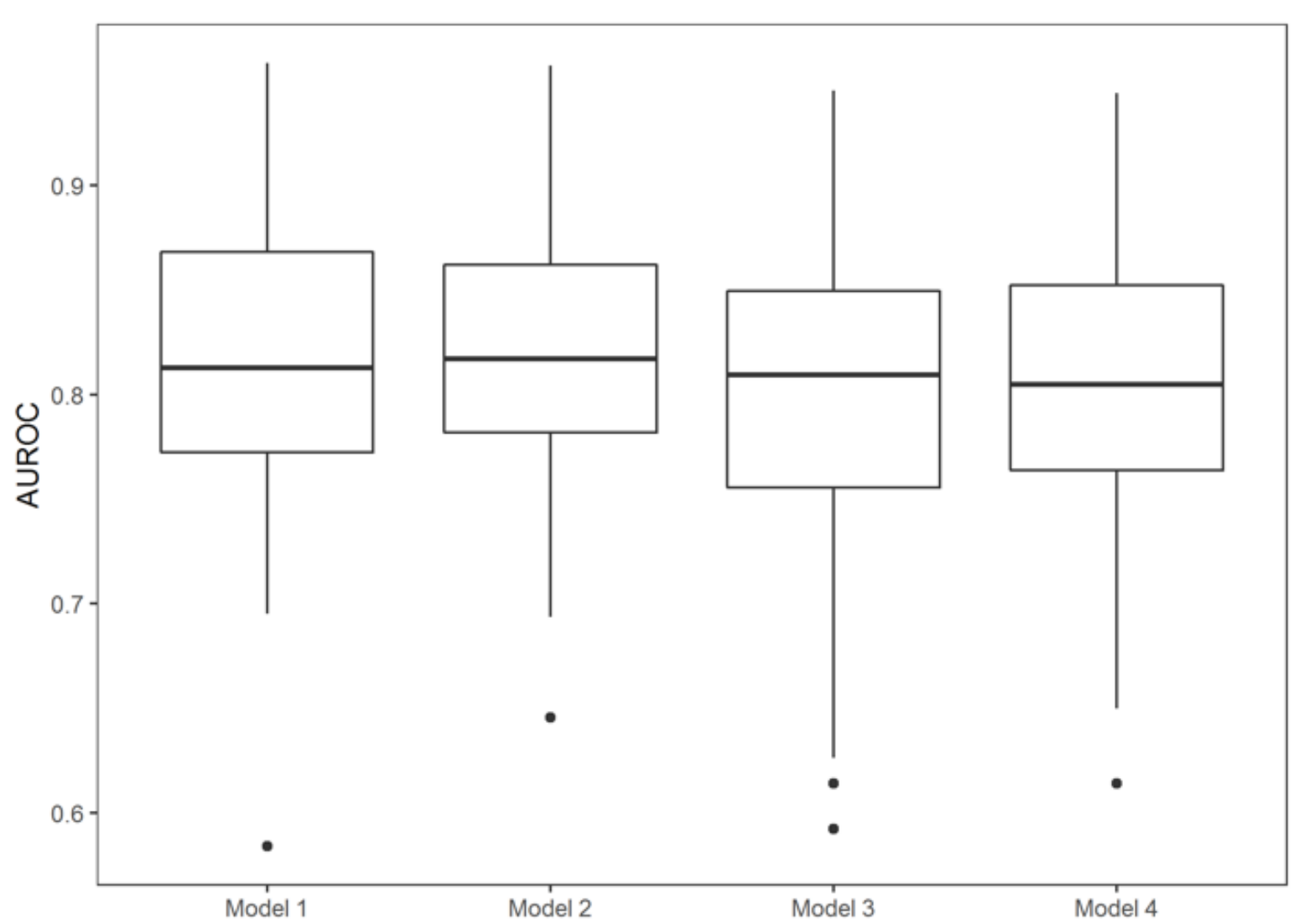

Figure S3. The distribution of AUROC from 10-fold cross-validation in the development set (with ten different randomizations) for the three potential plus-models and NORRISK2 (model 4) for women. AUROC area under the receiver operating characteristic. The Tromsø Study 2007-8.

Table S1. Evaluation of classification performance on the test set achieved by the different models. To calculate the measures, a cutoff of the continuous predicted values must be selected. Here they are selected to maximize the F1 score. O overall, W women, M men, ML machine learning. The Tromsø Study 2007-8.

Model	Sensitivity			Precision			Specificity			Accuracy			F1 score		
	O	W	M	O	W	M	O	W	M	O	W	M	O	W	M
ML 10 variables	0.22	0.05	0.31	0.16	0.05	0.20	0.95	0.97	0.92	0.92	0.95	0.89	0.18	0.11	0.24
ML 4 variables	0.28	0.26	0.29	0.10	0.08	0.12	0.90	0.92	0.87	0.87	0.90	0.84	0.15	0.12	0.17
ML 1 variable	0.35	0.37	0.34	0.08	0.06	0.10	0.82	0.84	0.81	0.81	0.83	0.78	0.13	0.10	0.15
Original NORRISK2	0.43	0.47	0.54	0.17	0.10	0.20	0.91	0.89	0.87	0.89	0.88	0.86	0.24	0.17	0.30
Recalibrated NORRISK2	0.43	0.26	0.57	0.17	0.11	0.19	0.91	0.94	0.85	0.89	0.92	0.84	0.24	0.16	0.28
Extended NORRISK2+	0.56	0.37	0.51	0.13	0.11	0.15	0.84	0.92	0.82	0.83	0.91	0.81	0.21	0.17	0.23
Combined model	0.28	0.32	0.51	0.20	0.12	0.17	0.95	0.94	0.85	0.93	0.92	0.84	0.23	0.17	0.26

Confusion matrices from the test set. The cutoffs used are selected to maximize the F1 score.

Table S2. Confusion matrix for the 10-variable machine learning model using a cutoff of 0.73.

	Positive	Negative	
CVD	12	42	54
No CVD	64	1223	1287
	76	1265	1341

Table S3. Confusion matrix for the 4-variable machine learning model using a cutoff of 0.61.

	Positive	Negative	
CVD	15	39	54
No CVD	131	1156	1287
	146	1195	1341

Table S4. Confusion matrix for the 1-variable machine learning model using a cutoff of 0.56.

	Positive	Negative	
CVD	19	35	54
No CVD	226	1061	1287
	245	1096	1341

Table S5. Confusion matrix for the original NORRISK2 model using a cutoff of 0.19.

	Positive	Negative	
CVD	23	31	54
No CVD	116	1171	1287
	139	1202	1341

Table S6. Confusion matrix for the recalibrated NORRISK2 model using a cutoff of 0.11.

	Positive	Negative	
CVD	23	31	54
No CVD	111	1176	1287
	134	1207	1341

Table S7. Confusion matrix for the extended NORRISK2+ model using a cutoff of 0.08.

	Positive	Negative	
CVD	30	24	54
No CVD	201	1086	1287
	231	1110	1341

Table S8. Confusion matrix for the combined model using a cutoff of 0.11.

	Positive	Negative	
CVD	15	39	54
No CVD	61	1226	1287
	76	1265	1341

*Table S9. Results from bivariate Fine-Gray analysis in the development set (pooled after imputation) for men. * p-value < 0.05.*

Variable	Subhazard ratio	Standard error	95% confidence interval	p-value
Pre SDNN	0.67	0.11	0.48-0.93	0.015*
Post SDNN	0.62	0.11	0.44-0.87	0.005*
Pre RMSSD	0.83	0.14	0.59-1.16	0.278
Post RMSSD	0.80	0.14	0.57-1.11	0.182
Pre PNN50	0.92	0.03	0.86-0.99	0.021*
Post PNN50	0.91	0.04	0.84-0.98	0.013*
Pre WAVD8	0.90	0.07	0.77-1.06	0.205
Post WAVD8	0.88	0.08	0.74-1.05	0.167
Pre BRS	1.09	0.09	0.94-1.27	0.263
Post BRS	0.99	0.08	0.85-1.16	0.914

*Table S10. Results from bivariate Fine-Gray analysis in the development set (pooled after imputation) for women. * p-value < 0.05.*

Variable	Subhazard ratio	Standard error	95% confidence interval	p-value
Pre SDNN	0.41	0.09	0.27-0.62	0.000*
Post SDNN	0.41	0.10	0.26-0.65	0.000*
Pre RMSSD	0.56	0.14	0.35-0.91	0.018*
Post RMSSD	0.56	0.14	0.34-0.91	0.018*
Pre PNN50	0.87	0.04	0.79-0.95	0.003*

Post PNN50	0.81	0.04	0.74-0.90	0.000*
Pre WAVD8	0.66	0.08	0.53-0.83	0.000*
Post WAVD8	0.69	0.08	0.54-0.87	0.002*
Pre BRS	1.32	0.13	1.09-1.59	0.004*
Post BRS	1.02	0.11	0.82-1.25	0.883

Supplementary Methods

Data filtering of hemodynamic measurements for prediction modeling

Hemodynamic measurements from the Finometer were used both in the statistical prediction model (NORRISK2+), and the machine learning approach. Spontaneous baroreceptor sensitivity was calculated by the sequence technique as described by Bruehl et al.[1]

We used Finometer recordings that had been extracted from the Finometer devices using the Beatscope software and stored in a Matlab file. These recordings contained the following hemodynamic variables from 9946 participants, with one measurement per detected heartbeat:

- Time
- reSYS
- reDIA
- reMAP
- LevelCor
- LevelCal
- Total
- HR
- IBI
- LVET
- SV
- CO

- TPR
- NoBeat5s
- Physiocal
- SpikeWave
- PseudoBeat
- Oscillations
- WaveformDamped
- Unknown
- Marker
- Height
- Zao
- Cwk
- Dist
- SPTI
- DPTI
- DPTI/SPTI
- RPP
- DPTI/RPP
- dp/dt
- Ext2

From these variables, the IBI was used in the calculation of HRV variables used in NORRISK2+, and the following ten variables were selected as candidate variables for the machine learning approach:

- reSYS
- reDIA
- reMAP
- HR
- IBI

- LVET
- SV
- Zao
- Cwk
- dp/dt

All of these variables, except HR and IBI, were corrected for the device recalibration periods (Physiocal) by replacing the values during recalibration by linear interpolation over these periods. In order to filter out measurement artifacts, the time-series for all of these variables were cleaned by an algorithm that detects artifacts based on distances to quartiles of a moving window algorithm, where detected artefacts were replaced by interpolated values[2]. In addition, and only for the PRV calculation, the RHRV library (that was used for PRV calculation) used an additional artefact filter function (FilterNIHR).

In order to extract relevant data intervals according to experimental phases, we used the phase label variable (Ext2) to extract IBI intervals from the pre-CPT (phase 2) and post-CPT (phase 5) periods for PRV calculation. The cumulative sum of IBI was used to generate the time array for calculation in RHRV 4.0. Only recordings with valid phase lengths according to the experiment were included, set to 26-31s for the pre-CPT interval and 59-62s for the post-CPT interval. For the machine learning approach, we extracted hemodynamic data from phase 2 (pre-CTP), phase 3 (prepare for CPT), phase 4 (CPT), phase 5 (post-CPT) and phase 6 (post-CPT baseline). Valid phase lengths were set to 26-31s (phase 2), 59-62s (phase 5) and 15-20s (phase 6).

Because we are using these data for prediction modeling at the individual level in this study, and that the Finometer measurement is susceptible to artifacts from technical errors, we aimed to also exclude recordings of low quality. To this end, all the recordings (with non-zero phase labels) were visually inspected by plotting the reSYS, reDIA and IBI time-series from phase 2 to phase 6 of the experiment, and recordings significantly disturbed by technical errors (recognized by non-physiological signal variations) were marked for exclusion. In addition, all IBI recordings corresponding to an SDNN>150 ms were visually checked for suspected errors and labeled for exclusion in such cases. We also used the frequency of the Physiocal recalibrations as an

indicator of measurement quality, marking any recording with a Physiocal frequency above 2 per minute for exclusion. Furthermore, the percentage of data points identified as IBI outliers by the Deegan algorithm or percentage of heartbeats rejected by the RHRV FilterNIHR algorithm within the pre or post CPT phases were used as the last quality indicators, where measurements with more than 20% in either algorithm were excluded. In summary, the following criteria were used for exclusion of measurements of low quality:

- Visual inspection (368 recordings)
- Physiocal per minute > 2.0 (759 recordings)
- RHRV FilterNIHR rejected beats >20% (62 recordings)
- Deegan outliers pre or post CPT > 20% (17 recordings)
- Extreme outliers in the level of systolic blood pressure, diastolic blood pressure or heart rate, identified by visual inspection (8 recordings)
- RHRV exclusion (15 recordings)

In total, this amounts to 1098 unique recordings for exclusion.

Based on the list of remaining IDs for each recording after exclusion, a list of IDs was created for use as a developement and test set for both the statistical prediction modeling and the machine learning approach. In order to use an identical set of IDs for both approaches, only the overlapping set of IDs were used for the analysis, totaling 6694 recordings.

References

1. Bruehl, S. *et al.* Chronic pain-related changes in cardiovascular regulation and impact on comorbid hypertension in a general population: The Tromsø study. *Pain* **159**, 119–127 (2018).
2. Deegan, Brian. M. T., O'Connor, M., Lyons, D. & ÓLaighin, G. A new blood pressure and heart rate signal analysis technique to assess Orthostatic Hypotension and its subtypes. in *Annual International Conference of the IEEE Engineering in Medicine and Biology - Proceedings* 935–938 (IEEE, 2007). doi:10.1109/IEMBS.2007.4352445.
